# Factors associated with undernutrition among children aged between 6–36 months in Semien Bench district, Ethiopia

**DOI:** 10.1016/j.heliyon.2021.e07072

**Published:** 2021-05-18

**Authors:** Dinaol Abdissa Fufa, Teshale Darebo Laloto

**Affiliations:** Department of Nutrition and Reproductive Health, School of Public Health, College of Health and Medical Sciences, Mizan-Tepi University, Mizan-Aman, Ethiopia

**Keywords:** Children aged between 6-36 months, Undernutrition, Semien Bench, Ethiopia

## Abstract

**Background:**

Malnutrition is a term used to refer a condition of both excessive and under-nutrition. Even in the 21^st^ c, it is yet among the major public health challenges that affect the health, growth, and development of millions of children across continents. Studies show that malnutrition during early childhood could result in devastating long-term effects such as poor school performance, weak immune system, and growth and development. Unfortunately, Ethiopia is among the developing countries hard hit by the problem of malnutrition (under-nutrition).

**Objective:**

To assess the magnitude of stunting, wasting, and underweight and risk factors associated with them; among Children aged between 6- 36 months.

**Methods:**

A community-based cross-sectional study design was conducted on 700 study participants from April to May 2020. Nutritional status of children aged between 6-36 months was determined based on the WHO reference population with Z score ≤ -2 SD (HAZ, WHZ and WAZ) was looked upon for stunting, wasting and underweight accordingly. Data were collected through structured and measuring anthropometric of the eligible sample unit. The questionnaire data were first entered into Epi-data and later analyzed following binary and multiple logistic regression analysis procedures with the help of IBM SPSS 26. Adjusted odds ratios, with 95% CI of the association and statistical significance declared at P-values ≤ 0.05 in this study.

**Results:**

In the present study, the response rate of the respondent was 100%. Factors significantly associated with undernutrition: stunting, Mothers who have no formal education (AOR = 2.58, 95% CI; 1.44–4.63), food insecure (AOR = 1.9, 95% CI; 1.23–2.9) and children had no feeding plate (AOR = 1.53, 95% CI; 1.07–2.19).

**For underweight:**

have not individual feeding plate (AOR = 2.39, 95% CI; 1.42–4.03), poor dietary diversity (AOR = 1.82, 95% CI; 1.23–2.69) and food insecurity (AOR = 4.24, 95% CI; 2.68–6.71).

We have also identified age between 6-11 months (AOR = 6.81, 95% CI; 2.93–15.79), 12–23 months (AOR = 2.28, 95% CI; 1.03–5.06), food insecure (AOR = 10.34, 95% CI; 5.22–20.45) and poor dietary diversity (AOR = 5.58, 95% CI; 2.36–13.19) as risk factors associated with wasting.

**Conclusion:**

This study relived that six variables significantly associate with undernutrition. These are: children have not his/her own feeding plate, household food insecurity, mother who had no formal education, poor dietary diversity and children aged between 6-11months and children age 6–23 months. Based on the findings of this study, the following recommendations are made. First, strategies and programs targeted towards the reduction and prevention of undernutrition among 6–36-month children should be made at all level to improve childhood nutritional status. Second, provide health information to families regarding the importance of separating children's feeding plate. Three, provide nutritional counselling about feeding practice and dietary diversity for mothers who have no formal education.

## 1 Introduction

Globally, malnutrition continuous to be a major public health challenge affecting the health, growth and development of children. Particularly young children in their 2–3 years of life are critically at greatest risk for malnutrition. Malnutrition is the common term for both excessive and under-nutrition. The causes of under-nutrition are insufficient consumption of nutrients or disrupted absorption of one or more nutrient [1]. Studies showed that the effects of undernutrition in early childhood often irreversible. For example, malnutrition in early childhood may affect growth and prevent children from achieving their optimum height/weight for age. Malnutrition during early childhood may also result in long term effects such as poor school performance and weak immune system [2, 3].

Worldwide, based on Food and Agriculture Organization of the United Nations report, over 690 million (8.9%) people were undernourished in 2020. In the same year it is documented that around 49 million (21.9%) children were stunted and about 49.5 million (7.3%) children were wasted. In Africa, the magnitude of stunting and wasting were reported to be 39% and 28% respectively [4, 5, 6, 7]. The severity of undernourishment in Africa (17.6%) was observed to be twice than the world (8.9%) [4].

In Ethiopia, different studies reported different magnitude for stunting, wasting and underweight ranging from 14% to 66.6%, 2%–21%, and 5%–32% respectively [8, 9]. A recent estimate based on the 2019 mini Ethiopian Demographic Health Survey (EDHs), indicated the national prevalence of stunting, wasting, and underweight were 37%, 7% and 21% respectively. The trends of malnutrition among children in Ethiopia is decreasing at a lower rate. For example, the magnitude of stunting in 2000 was 52% compared to 37% in 2019; and wasting in 2000 was 11% compared to 7% in 2019. However, undernutrition yet remains a public health concern as reported by revealed WHO [6, 8, 10].

Major root causes of under-nutrition are several and complicated, these causes are connected and hierarchically joined to each other. The immediate causes are insufficient diet habit, child diarrhoea, and ages of breastfeeding children; that are themselves caused by a collection of underline factors. Underlying cause includes: household dietary diversity; age of introduction of complementary food; access to safe water and environmental hygiene. These underlying factors are also influenced by the basic socio-economics and political conditions [11]. Malnutrition involves a wide range of socio-economic and demographic factors such as household's financial condition, income, residence, occupation, education, maternal age and family size, personal and environmental hygiene and infection [12].

Evidence shows that intervening malnutrition in early childhood has a significant impact in later life. For instance, investing 10 dollars in nutrition per child every year could prevent 3.7 million child death and 65 million children from stunting [13, 14]. An appropriate intervention requires a proper understanding of magnitude and the underlying causes of the problem across different contexts. To this end, to the best of researchers’ knowledge, there were few studies conducted regarding early childhood malnutrition in Ethiopia. Even these few studies were conducted to assess exclusively wasting [15]. Hence, they did not provide a full picture of the extent of malnutrition in early childhood. Therefore, the present study was aimed to examine all forms of undernutrition (wasting, stunting and underweight) among children aged between 6-36 months.

## 2 Methods

### 2.1 Study area, design and period

A community-based cross-sectional study design was conducted from April to May 2020 in Semien Bench district. Semien Bench district is one of the 8 districts in Bench Sheko Zone, Southern Nation, Nationalities, and Peoples Region of Ethiopia.

Livelihoods of the population in the district predominantly depends on cultivating crops and breeding livestocks [16]. Most of the inhabitants were Protestants, with 64.28% of the population reporting that belief, 19.29% practised traditional beliefs, and 6.58% practised Ethiopian Orthodox Christianity. The total estimated population size of the district is 159,480: of which 87,748 (51.26%) were female and 77,732 (48.74%) were male; 6507, were urban and 152,973 were rural population residents [17]. There are 31 kebeles (Kebele is the smallest administrative unit in Ethiopia) in Semien Bench district where 28 kebeles are rural and 3 of them are urban. The estimated total number of children <36 months in the district is 17,200. Regarding the health service coverage in the area: there are 31 health post and four health Center.

### 2.2 Source of data and study population

The source populations were mothers/caregiver with child pairs aged between 6-36 months and who lived at least for over six months in the study area. The study population were all randomly selected children aged between 6–36 months who were living with their mothers/caregivers in selected kebele.

### 2.3 Eligibility

**Inclusion Criteria:** All children aged between 6-36 month and their mothers or caregivers who were living for at least 6 months in the study area.
**Exclusion Criteria:** All children aged between 6-36 month and their mothers or caregivers who were critically ill during the time of data collection and children had physical deformities of limbs and spines.

### 2.4 Sample size calculation

The required sample size was determined using double population proportion formula by using STAT CALC application of EPI- INFO version 7 software. The sample size determination had considered the following assumptions: Two-sided confidence level of 95%; Power of 80; 0.5 margins of error; and 10% of contingency non-response rate. .The exposed variables were paternal educational level, child sex and occurrence of diarrhoea in the last two weeks. Ultimately, the largest sample size was taken (636 + 63.6–700) [18,15,19]. Proportionate sample sizes were also calculated for each kebele.

### 2.5 Sampling procedures

Of the total 31 kebeles, 10 kebeles were selected by using simple random sampling technique.

To develop a sampling frame, we did a census in the selected kebeles. Before starting the study, enumeration of households having children aged between 6-36 months was carried out strictly following the eligibility criteria. The households that only fulfilled the inclusion criteria were consider as study units. We distributed the sample size to each kebele concerning to the total sample size for the study.

By using the simple random sampling method the first household was selected and the remaining were selected systematically at the interval of K. Interval (K value) was determined for each kebele by dividing the total eligible children in the kebele to the sample proportion. Whenever over one eligible children were encountered in a given household, one child was randomly chosen by a lottery method.

### 2.6 Data collection procedures and instruments

We collected the data by ten trained diploma nurses. Two days training was given to the data collectors before starting the data collection on how to take anthropometric measurements, interview, selection criteria of households, and how to approach the respondents. We collected data through a home-to-home visit by using structured and pretested questioners and anthropometric measurements. The questionnaire was adapted from different literatures and guidelines [8, 15, 20, 21]. It includes; Socio-Demographic, Household Wealth Index, Household Food Security Access Scale (HFIAs), Minimum Dietary Diversity (MDDs) and nutritional status of the children.

The questionnaire was initially developed in English language and translated into Amharic which is the official language in the study area by a language expert. Then, to check its consistence, the questionnaire was translated back to the English language by an individual who was blind to the original version and fluent in English and local languages. Data on date of birth of children were gathered from written birth certificates or immunization cards. Whenever such written documents were not available, data given by mothers or caregivers was used instead and crosschecked from the family folder.

To measure household wealth, Household Wealth Index was scored based on the number and kinds that the household goods they have, ranging from hen to oxen in addition to the characteristic of housing, like flooring material, latrine facility, source of drinking water and home environment. Then, by using principal component analysis, we computed the composite score. Finally, the household wealth quintiles were compiled by assigning the household score to each household, ranking each household in the community by their score and dividing the distribution into three equal categories; poor, middle and rich [15]. To determine household food security status, nine standard questions of the Household Food Insecurity Access Scale (HFIAs) were used that were developed for this purpose by Food and Nutrition Technical Assistance (FANTA) in 2007. Each respondent was asked the amount and variety of eaten food, and occurrence of food shortage for the household, causing them not to eat the whole day or eat at night only in the past four weeks before the survey [21]. All yes responses of the individual were coded as one and no response of the individual was coded as zero and finally, the responses of all individuals were summed to produce a household food insecurity index. The index had internal consistency (Cronbach alpha = 0.90). Then, food security was coded as one and food insecurity were coded as zero.

Children Minimum Dietary Diversity (MDDs) status was determined based on the proportion of children who were feed less than four food groups and four and more food groups out of seven food groups. It was categorized into two dietary intakes less than four food groups and four or more food groups which were coded as zero and one respectively. The children who were fed less than four of the major food group were considered to have poor dietary diversity [20].

### 2.7 Anthropometric measurement

**Length or height** was measured (model No; S0114520 which was manufactured by UNICEF for this purpose) without shoes, socks, hair/headscarf, and ornaments and positioning the subject at the Frankfurt plane by using horizontal or vertical movable wooden board measured to the nearest 0.1 cm. The heels, buttocks, scapulae, and head are positioned in contact with the horizontal or vertical backboard [22].

**Weight** was measured by the spring hanging scale (model No; s0189000 which was manufactured by UNICEF for this purpose), Hook the scale to a tripod or a stick held Weight was measured by the spring hanging scale, Hook the scale to a tripod or a stick held horizontally by two people at eye level. Suspend the weighing pants from the lower hook of the scale and readjust the scale to zero before measuring. The child is dressed in a set of plastic, then the child hangs freely without holding onto anything and measured to the nearest 0.1 Kg [22].

**Middle upper arm circumference: was** measured by the MUAC tape made from synthetic paper (model No; S0145620 which was manufactured by UNICEF for this purpose). We removed any clothing that may cover the child's left arm then calculated the midpoint of the child's left upper arm by first locating the tip of the child's shoulder, Bend the child's elbow to make a right angle, and Inspected the tension of the tape on the child's arm. We also made sure that the tape has proper tension and not too tight or too loose. When the tape was in the correct position on the arm with the correct tension, read and called out the measurement to the nearest 0.1cm [22, 23].

### 2.8 Data quality control

To assure the quality of the data: the questionnaire was pretested on 5% of the study sample size where the cases were from kebeles not incorporated in this study; two days training was giving for data collectors on the instruments, method of data collection, how to take anthropometric measurements, ethical issues and the purpose of the study . To minimize random anthropometric measurement error the intra and inter-observer's variability of the data collector's relative technical error of measurement (%TEM) was calculated during training among 10 children age between 6-36 Months. The accepted relative technical measurement errors for intra-observers was less than 1.5% while for inter-observers was less than 2%. Data collectors' accuracy of anthropometric measurements was standardized with their trainer during training and pretesting [24]. Data collectors were measured at least two separate anthropometric measurements. Double data entry was done to make comparisons of two data cells and resolve whenever there was some difference.

### 2.9 Data processing and analysis

The collected data was checked for its completeness and consistency during data collection on daily bases. Then each completed questioner was assigned a unique code and double entered on to Epi Data 4.6.02 software and cleaned for implausible and missed values accordingly. The nutritional status of children was generated by using the WHO Anthro version 3.1.0 software. For further analysis, the data were exported to SPSS version 26 software. Descriptive analyses were carried out to explore the socio-demographic characteristics of the respondents by using frequency tables.

The outcome variable was recoded to dichotomous outcomes as undernourished or not. Stunted, underweight and wasted Children Age between 6-36 Months Z score < -2SD was coded as '1′ and those with normal Z score was coded '0'. The independent variables were coded based on previous related studies and the distribution of responses in the data [15, 25].

To measure household food insecurity, household food insecurity access scale was used to identify whether the household was food insecure or not [26].

The children's minimum dietary diversity (MDD) were categorized based on WHO's “Indicators for assessing infant and young child feeding practices” guideline as poor dietary diversity or good dietary diversity [20]. The family wealth index was constructed by using a Principal Component Analysis (PCA) method considering locally available household assets into terciles and the family wealth was categorized accordingly.

The multicollinearity effect was checked by looking at the standard error and non-collinear covariates were included in the independent binary logistic regression model to assess the possible association between each independent variable and outcome variable. Those variables with a P-value of ≤0.25 during bivariable analyses were chosen for subsequent analyses by using multiple logistic regressions to control for all possible confounders and to identify factors associated with stunting, wasting and under-weight (outcome variable). The fitness of the model was tested by Hosmer- Lemeshow goodness of fit test. Adjusted odds ratios along with 95% CI of the association and statistical significance was declared at P values ≤0.05 in this study.

### 2.10 Operational definition

**Wasting: -** Children aged between 6-36 months who her/his weight-for- height is below the –2 z-score line [27].

**Stunting: -** Children aged between 6-36 months who her/his length/height-for-age is below the –2 z-score line [27].

**Under weight:** - Children aged between 6-36 months who her/his weight-for- age is below the –2 z-score line [27].

### 2.11 Ethical approval

This study was conducted following the declaration of Helsinki. Ethical clearance was obtained from Mizan-Tepi University Institutional Health Research Ethics Review Committee (IHRERC) IRB 06847/2020. A permission letter was obtained from Mizan-Tepi University and submitted to the Semien Bench District health office. Informed voluntary written and signed consent and assent was obtained from each child's mother or caregiver after explaining the study, purpose, procedure and duration, possible risks and benefits of the study. The consent process of this study was approved by IHRERC.

## 3 Results

### 3.1 Socio-demographic characteristics and economic status of the respondents

A total of 700 study participants were sampled for the study. the response rate was 100%. The majority (97%) of mothers or caregivers who participated in the study were married. The mean age ±standard deviation of mother/caregivers were 33 ± 4 years old. Regarding maternal educational status, 637 (91%) of mothers/caregivers had no formal education and near to two-third of the mothers were housewife. The mean age ±standard deviation of the children was 23.38 ± 9.3. Concerning age category of the children, 86 (12.3%) were between 6-11 months, 189 (27%) were between 12-23 months and 425 (60.7%) were between 24-36 months. The household wealth index shows that approximately half of the sample households were poor and 33 % had middle socio-economic positions (Table 1).

**Table 1:** Socio-demographic characteristics and the economic status of the respondent in Semien Bench district, Ethiopia, 2019.

Variable	Category	Frequency	Per cent
Age of mothers	15–24	151	21.57%
	25–34	267	38.15%
	35–44	177	25.28%
	46–54	105	15%
Religion	Protestant	413	59%
	Orthodox	189	27%
	Muslim	77	11%
	Others	21	3%
Paternal education status	Illiterate	426	67.9%
	Literate	218	32.1%
Paternal occupational status	Farmer	669	98.4%
	Other	11	1.6%
Family size	<5	251	37%
	≥5	441	63%

Children from mothers who had no formal education (94.7%) were more stunted compared to children from mothers who had formal education (5.3%) with p = 0.001. Similarly, the severity of under-weight was more common (94.9%) among children from mothers who had no formal education compared to children from mothers who had formal education (5.1%) with p = 0.049.

From the total (342) stunted children, 180 (52.6%) were consuming less than four food groups and others consumed four and above food groups 162 (47.4%) with p = 0.0001. The degree of under-weight was high 105 (66.3%) among children who consumed less than four food groups as compared to children who had consumed four and above food groups 53 (33.5%) with p = 0.0001. The magnitude of wasting was also high 40 (85.1%) in children who consumed less than four food groups as compared to children who had food groups four and above 7 (14.9) with p = 0.001.

### 3.2 Children characteristics and caring practice

The mean age ±standard deviation of children was 23.38 ± 9.3 months old. Concerning length/height, the mean length/height ±standard deviation of the children was 79.16 ± 8.5. The mean MUAC ±standard deviation of the children was 13.85 ± 1.18. Regarding nutritional status: stunting, under-weight, and wasted among children aged between 6-11 months was 27(7.9%), 25(15.8%) and 17 (36.2%) respectively. On the other hand, stunting, under-weight and wasted among children between 12-23 months was 95(27.8%), 46(29.1%) and 14 (29.8%) respectively. Similarly 24–36 months children were 220(64.3%), 87(55.1%) and 16 (34%) belongs to the nutritional status of the children stunting, under-weight and wasted respectively (Table 2).

**Table 2:** Children aged between 6-36 months characteristics and caring practice in Semien Bench district, Ethiopia, 2019.

Variable	Category	Frequency	Per cent
Child age	6–11 months	86	12.3%
	12–23 months	189	27%
	24–36 months	425	60.7%
Child sex	Male	350	50%
	Female	350	50%
Birth interval	≤2 years	107	15.28%
	>2 years	593	84.71%
Exclusive breastfeeding	<6 months	229	32.7%
	At 6 months	471	67.3%
Diarrhoea during last 2 weeks	Yes	73	10.42%
	No	627	89.57%

### 3.3 Minimum acceptable dietary diversity

Out of the total children, 343 (49%) met the minimum acceptable dietary diversity. Majority (81%) of the children had received foods from fruits, legumes and staple food. Around 25% of them have consumed milk and milk products before 24 h of the data collection (Table 3).

**Table 3:** Children aged between 6-36 months dietary diversity practice in Semien Bench district, Ethiopia, 2019.

Food group	Frequency	Per cent
Grains, roots or tubers	462	66%
Vitamin A-rich plant foods	357	51%
Other fruits or vegetables	420	60%
Meat, poultry, fish, seafood	49	7%
Eggs	70	10%
Pulses/legumes/nuts	490	70%
Milk and milk products	175	25%

### 3.4 Risk factors associated with stunting among children aged 6–36 months

Some variables that were associated during bivariate analysis model had showed no significant association during multiple variable analysis model. The following three variables (children had no individual feeding plate, household food insecurity and mothers had no formal education) were significantly associated with stunting in the multiple variable analysis model at P < 0.05.

In the current study, the maternal educational level was associated with childhood stunting. Children whose mothers had no formal education were 2.58 times (AOR = 2.58, 95% CI; 1.44–4.63) more likely to be stunted compared to children whose mothers had formal education. Similarly, household food insecurity was associated with childhood stunting. Children from food insecure household were 1.9 times (AOR = 1.9, 95% CI; 1.23–2.9) more likely to be stunted as compared to children from food secure household. Similarly, children who had no separate feeding plate also showed association with childhood stunting. Children who had no separate feeding plate were 1.53 times (AOR = 1.53, 95% CI; 1.07–2.19) more likely to be stunted as compared to their counterpart (Table 4).

**Table 4:** Both bivariate and multiple logistic stunting of children aged between 6-36 months dietary diversity practice in Semien Bench district, Ethiopia, 2019.

Variable	Stunting		COR 95% CI	AOR 95% CI
	Yes	No		
**The child had a separate feeding plate**				
No	268	252	1.52 (1.08–2.15)	1.53 (1.06–2.18)
Yes	74	106	1	1
**Decision - making strategy of the sick child**				
Individual	106	89	1.36 (.97–1.89)	1.29 (0.92–1.82)
Jointly	236	269	1	1
**Child sex**				
Male	182	168	1.28 (0.95–1.73)	1.23 (0.90–1.67)
Female	160	190	1	1
**Household Food security**				
Food insecure	68	46	1.68 (1.12–2.53)	1.88 (1.22–2.89)
Food secure	274	312	1	1
**Children age**				
6–11 months	27	59	0.45 (0.26–0.67)	0.41 (0.24–0.67)
12–23 months	95	94	0.94 (0.67–1.33)	0.95 (0.67–1.36)
24–36 months	220	205	1	1
**Maternal education**				
Illiterate	324	313	2.59 (1.47–4.57)	2.58 (1.44–4.62)
literate	18	45	1	1

### 3.5 Risk factors associated with underweight among children aged 6–36 months

Several variables that showed significant associated during binary logistic regression analysis model did not make association during multiple Logistic Regression analysis. But most of these variables did not make significant association during multiple logistic regression analysis. Only the following three variables (have not individual feeding plate, poor dietary diversity, and food insecurity) were significantly associated with under-weight in the multiple variable analysis model at P < 0.05.

In this study, children have no separate feeding plate has associated with under-weight. Children who had no separate feeding plate were 2.39 times (AOR = 2.39, 95% CI; 1.42–4.03) more likely to be under-weight as compared to children who had separate feeding plate. Similarly, children who had poor dietary diversity has showed significant association. Children who had consumed less than four of the major food groups in the last 24 h prior to data collection were 1.82 times (AOR = 1.82, 95% CI; 1.23–2.69) more likely to be under-weight as compared to their counterparts. Household food insecurity also showed significant association with children under-weight. Children from food insecurity household were 4.24 times (AOR = 4.24, 95% CI; 2.68–6.71) more likely to be under-weight as compared to children from food secured household (Table 5).

**Table 5:** Both bivariate and multiple logistic undernutrition of children aged between 6-36 months dietary diversity practice in Semien Bench district, Ethiopia, 2019.

Variable	Undernutrition		COR 95% CI	AOR 95% CI
	Yes	No		
**The child had a separate feeding plate**				
No	134	386	2.25 (1.41–3.62)	2.39 (1.42–4.00)
Yes	24	156	1	1
**Child sex**				
Male	87	263	1.30 (0.91–1.85)	1.17 (0.80–1.71)
Female	71	279	1	1
**Household Food security**				
Food insecure	51	63	3.62 (2.37–5.54)	4.24 (2.67–6.71)
Food secure	107	478	1	1
**Maternal education**				
Illiterate	150	487	2.12 (0.98–4.54)	1.88 (0.84–4.20)
literate	8	55	1	1
**Children age**				
6–11 months	25	61	1.59 (0.94–2.68)	1.53 (0.87–2.66)
12–23 months	46	143	1.25 (0.83–1.88)	1.24 (0.80–1.90)
24–36 months	87	338	1	1
**MDD 24 h before the survey**				
<4 food items	105	252	2.28 (1.57–3.30)	1.82 (1.22–2.69)
≥4 food items	53	290	1	1

### 3.6 Risk factors associated with wasting among children aged 6–36 months

Many variables that showed significant association during binary logistic regression analysis have not showed similar result during multiple logistic regression analysis. Only the following three variables (child age, poor dietary diversity and food insecurity) were significantly associated with under-weight in the multiple variable analysis model at P < 0.05.

Based on the finding of this study, children's age had associated with wasting. Children aged between 6-11 months were 6.81 times (AOR = 6.81, 95% CI; 2.93–15.79) more likely to be wasted compare to their counterpart. Similarly, children aged between 12-23 months were 2.28 times (AOR = 2.28, 95% CI; 1.03–5.06) more likely to be wasted compare to their counterpart.

Upon the finding of this study, household food insecurity had an association with wasting. Children from food insecure household were 10.34 times (AOR = 10.34, 95% CI; 5.22–20.45) more likely to be wasted compare to children from food secured household. Similarly, poor dietary diversity had an association with wasting. Children who consumed less than four of the major food groups in the last 24 h prior to data collection were 5.58 times (AOR = 5.58, 95% CI; 2.36–13.19) more likely to develop wasting compare to their counterpart (Table 6).

**Table 6:** Both bivariate and multiple logistic wasting of children aged between 6-36 months dietary diversity practice in Semien Bench district, Ethiopia, 2019.

Variable	Wasting		COR 95% CI	AOR 95% CI
	Yes	No		
**Children age**				
6–11 months	17	69	6.29 (3.04–13.05)	6.80 (2.93–15.93)
12–23 months	14	175	2.05 (0.97–4.28)	2.28 (1.03–5.06)
24–36 months	16	409	1	1
**Household Food security**				
Food insecure	28	86	9.71 (5.20–18.16)	10.33 (5.22–20.45)
Food secure	19	567	1	1
**MDD 24 h before the survey**				
<4 food items	40	317	6.06 (2.67–13.72)	5.58 (2.36–13.19)
≥4 food items	7	336	1	1
**Maternal education**				
Illiterate	45	592	2.38 (0.55–9.71)	1.96 (0.43–8.95)
literate	2	61	1	1

## 4 Discussion

Undernutrition at early childhood has a greater risk for illness, delayed mental development, poor school performance, and reduced intellectual capacity and even death. This study was conducted to determine the magnitudes of Undernutrition (wasting, stunting, and underweight) and its associated factors among children aged between 6-36 months. The finding of this study showed that the overall prevalence of stunting is 48.9 ± 0.5% (95 % CI: 48.4, 49.4). Based on WHO category for public health significance Cut-off values, the magnitude of stunting in the current finding is very high [6].

The total prevalence of underweight was 22.6% ± 0.4 (95 % CI: 22.2, 23). This indicates that there is a high prevalence of underweight children in the study area based on public health significance Cut-off values of WHO [6]. Furthermore, the overall prevalence of wasting was 6.7% ± 0.25 (95 % CI: 6.45, 6.95). This magnitude is categorized under; poor, based on public health significance Cut-off values of WHO [6].

The magnitude of stunting in the present study is higher than studies conducted in rural Ethiopia, Western Uganda, Brazil and Amazon [28, 29, 30, 31, 32]. The possible explanation for the difference could be environmental, behavioral characteristics of households and socio cultural condition.

The magnitude of stunting is similar with some of the studies conducted in India, [33]. The current finding is a little higher than the magnitude of underweight of the national prevalence [8]. The finding is also higher than some of the studies conducted in Amazon, China [28, 34]. The discrepancy might be due to socio-cultural difference like child feeding practice and health care seeking practice.

The magnitude of wasting in the present study is in line with the national prevalence of wasting in Ethiopia and in Vietnam [8, 32]. In the current study, the finding of wasting is lower than some of the studies conducted in Afar region, northeast Ethiopia, rural Ethiopia, Kemba Woreda, Southern Ethiopia, and Niger [25, 31, 35, 36]. The possible reason for the difference could be the sample size, exclusion of children less than 6 months and cultural difference. Moreover, it was higher than a study in China [34].

In the current study, all forms of undernutrition such as (stunting, wasting, and undernutrition) were found significantly associated with household food insecurity. Children from food insecure household were more likely to be wasted, stunted, and underweight than children from food secured household. This finding is in line with some of the studies conducted in India, Indonesia, South Africa and South Ethiopia [33, 37, 38, 39, 40]. This might be due to children from food insecure household could not access enough food intake and reduced dietary diversity, that may results in malnutrition in general.

Children who had no separate feeding plate showed significant association with stunting and underweight. This finding is supported by WHOs, the guideline for Infant and young child feeding and USAID, Counseling Guide for Complementary Feeding for Children 6–23 Months, which recommends that a child should have his or her own plate during feeding to combat malnutrition in general [41, 42].

Similarly, maternal educational level also associated with stunting. Children from mothers who had no formal education were more likely to be stunted than children from mothers had formal education. The finding of the present study is in line with studies conducted in; Ethiopia, Sub-Saharan Africa and India [43, 44, 45, 46]. This might be due to the fact that mothers who had formal education could have prior knowledge regarding child nutrition, hygiene behavior, and health-seeking behavior for childhood illnesses that reduce stunting.

Children's poor dietary diversity was also found significantly associated with underweight and wasting. Children who consumed less than four of the major food groups, out of seven food groups were more likely to develop under-weight and wasting as compared to children who consumed four and above of the major food groups. This finding is comparable with the evidence from other similar studies conducted in Ethiopia, Tanzania and India [47, 48, 49]. This is probably because of the fact that inadequate balancing diet leads to macro-and micronutrient deficiency, which is often serious during infancy and early childhood, and even might extent to adulthood.

In the current study, we observed that children's age was associated with wasting. Children aged between 6-11 months and 12–23 months were more likely to be wasted compare to others. This is in line with a study conducted in Northern Ethiopia, [44].

## 5 Limitation of the study

Due to the nature of a cross-sectional study design, it is difficult to establish a cause-effect relationship between the predictors and outcome variable. Social desirability bias especially related to household wealth index might affect the accuracy. Recall bias is also potential limitation that might have affected the accuracy of the information, especially related to 24 h dietary group recall and 30 days household food insecurity recall.

## 6 Conclusion

In the current study, some factors are associated with wasting (children aged between 6-36 months and children age 6–23 months, poor dietary diversity, and food insecurity), Underweight (have not individual feeding plate, poor dietary diversity, and food insecurity) and Stunting (children have no individual feeding plate, household food insecurity and mothers had no formal education). The strategies and program targeted to reduction, and prevention of undernutrition on children aged between 6-36 months should be made at all levels to improve childhood nutritional status, separate children feeding plate, provide nutritional counselling for mothers who had no formal education regarding to children feeding practice and dietary diversity.

## Declarations

### Author contribution statement

Dinaol Abdissa, Teshale Darebo: Conceived and designed the experiments; Analyzed and interpreted the data; Wrote the paper.

### Funding statement

This research did not receive any specific grant from funding agencies in the public, commercial, or not-for-profit sectors.

### Data availability statement

Data included in article/supplementary material/referenced in article.

### Declaration of interests statement

The authors declare no conflict of interest.

### Additional information

No additional information is available for this paper.
